# An Unexpected Intraoperative Discovery: A Parasitic Leiomyoma in a Perimenopausal Patient With Complex Abnormal Uterine Bleeding

**DOI:** 10.14740/jmc5254

**Published:** 2026-02-02

**Authors:** Kellee Diaz, Rahul Lohana, Manuel A. Navas

**Affiliations:** a Department of Clinical Medicine, Burrell College of Osteopathic Medicine, Las Cruces, NM, USA

**Keywords:** Parasitic leiomyoma, Abnormal uterine bleeding, Robotic-assisted hysterectomy, Minimally invasive, Invasive gynecologic surgery

## Abstract

Parasitic leiomyomas are an uncommon subtype of extra-uterine smooth muscle tumors characterized by complete detachment from the uterus and subsequent development of an autonomous vascular supply. Their atypical anatomical locations, independent blood flow, and frequent coexistence with large fibroid uteri make preoperative diagnosis difficult. Abnormal uterine bleeding (AUB) is one of the leading indications for hysterectomy worldwide, and leiomyomas remain the most common structural cause within the International Federation of Gynecology and Obstetrics (FIGO) PALM–COEIN classification. Although hysterectomy is considered curative for symptomatic fibroid disease, unexpected intraoperative findings, such as parasitic leiomyomas, can significantly alter operative planning, increase surgical complexity, and necessitate careful dissection to prevent bowel or vascular injury. We report the case of a patient who underwent robotic-assisted total laparoscopic hysterectomy with bilateral salpingectomy for symptomatic AUB and a markedly enlarged multi-fibroid uterus. During surgery, a distinct parasitic leiomyoma was discovered, densely adherent to the large intestine with no connection to the uterus. The mass had mesenteric-derived vascularity and required meticulous adhesiolysis for safe mobilization. The uterus and parasitic mass were delivered vaginally. Pathology confirmed benign intramural leiomyomas and separately submitted benign smooth muscle fragments consistent with parasitic leiomyoma. No atypia, necrosis, or abnormal mitotic activity was present. The patient recovered well and reported significant symptom relief at the 1-week postoperative visit. This case shows how difficult it can be to diagnose parasitic leiomyomas and why careful assessment is needed during hysterectomy for large fibroid uteri. We also discuss their causes, imaging challenges, surgical management, and follow-up to help inform clinicians.

## Introduction

Leiomyomas, including rare extrauterine variants, are the most common benign tumors of the female reproductive tract and remain one of the leading indications for hysterectomy worldwide [1–3]. These smooth muscle neoplasms arise from monoclonal proliferation within the myometrium and may result in abnormal uterine bleeding, pelvic pressure, pain, infertility, and pregnancy complications [2–4]. Although leiomyomas are typically confined to the uterus, several atypical variants exist, including parasitic leiomyomas [5]. Parasitic leiomyomas are defined by their complete separation from the uterus, reimplantation onto peritoneal or visceral structures, and establishment of an independent vascular supply [5, 6]. These tumors have been described on the bowel, pelvic sidewall, bladder, broad ligament, and omentum [5–7]. Two primary mechanisms have been proposed for their development. The classical mechanism involves torsion and ischemic detachment of a pedunculated subserosal leiomyoma, followed by reimplantation and neovascularization [8]. An iatrogenic mechanism has also been described, whereby tissue dissemination during laparoscopic morcellation results in implantation of leiomyoma fragments throughout the peritoneal cavity [6, 9, 10]. Although morcellation-associated parasitic leiomyomas have gained increased recognition, spontaneous cases continue to occur in patients without prior morcellation or abdominal surgery [8]. Preoperative diagnosis remains challenging. Imaging studies often cannot reliably distinguish parasitic leiomyomas from adnexal masses or gastrointestinal tumors, particularly in the presence of a large fibroid uterus [7, 11, 12]. Magnetic resonance imaging offers improved soft tissue characterization but may still fail to clearly identify extrauterine implantation [7, 11, 12]. As a result, parasitic leiomyomas are frequently diagnosed intraoperatively [5, 6]. In this report, we describe the incidental discovery of a parasitic leiomyoma adherent to the large intestine during robotic-assisted hysterectomy and discuss diagnostic challenges, treatment considerations, molecular mechanisms, and surgical implications relevant to clinical practice.

## Case Report

A 49-year-old woman presented with a 3-year history of progressively worsening abnormal uterine bleeding accompanied by severe dysmenorrhea and symptomatic anemia. Her menstrual cycles were regular every 28–30 days but had become increasingly heavy, requiring 10–12 tampons or maxi pads daily with frequent passage of large clots. Associated symptoms included fatigue, exertional dyspnea, dizziness, pelvic pressure, early satiety, intermittent constipation, and urinary frequency. Her medical history was notable for stage III chronic kidney disease, recurrent deep vein thromboses, type 1 diabetes mellitus, hypertension, hyperlipidemia, chronic iron-deficiency anemia, gastroparesis, and longstanding uterine leiomyomas. Past surgical history included gastric bypass, bilateral tubal ligation, appendectomy, cholecystectomy, and abdominal hernia repair. She denied tobacco, alcohol, or recreational drug use. There was no personal or family history of gynecologic malignancy, and cervical cancer screening was up to date. Physical examination revealed an enlarged, anteriorly positioned uterus consistent with a fibroid uterus. Laboratory evaluation demonstrated chronic anemia with a hemoglobin level of 10 g/dL and ferritin of 15.3 ng/mL. Pelvic ultrasonography revealed a markedly enlarged uterus measuring 17.2 × 8.9 × 12.0 cm with multiple intramural and subserosal leiomyomas. Both ovaries appeared normal with preserved Doppler flow. Endometrial biopsy demonstrated disordered proliferative endometrium without hyperplasia or malignancy. Given the patient’s significant symptom burden, chronic anemia, large multi-fibroid uterus, and failure of medical management, she elected to proceed with robotic-assisted total laparoscopic hysterectomy with bilateral salpingectomy.

### Intraoperative findings

**Figure 1:**
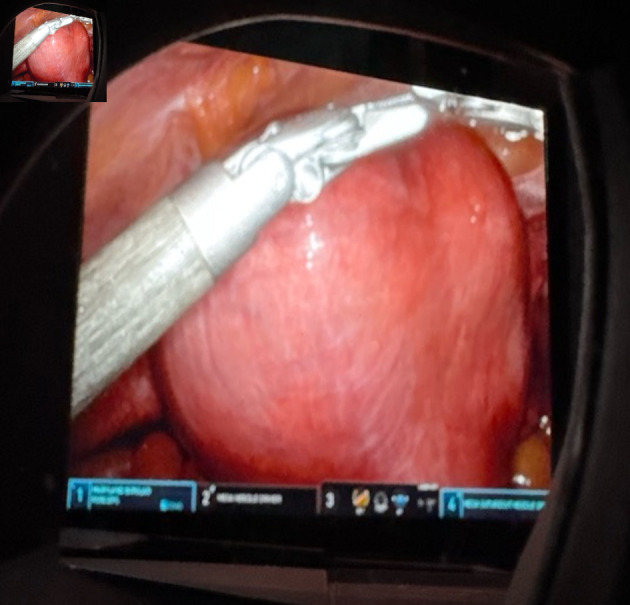
Intraoperative image of the markedly enlarged myomatous uterus prior to hysterectomy, demonstrating global fibroid involvement and distortion of pelvic anatomy.

**Figure 2:**
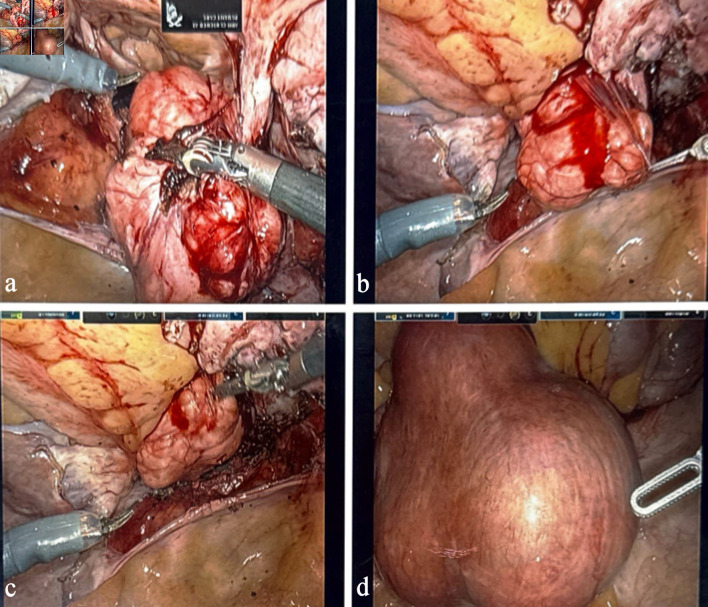
Intraoperative images demonstrating the parasitic leiomyoma (a–d). Panels show the mass adherent to the large intestine, its mesenteric vascular supply, and sequential adhesiolysis performed to safely mobilize the lesion during robotic-assisted hysterectomy.

Upon entry into the abdomen, the uterus was noted to be markedly enlarged, smooth, and globally myomatous (Fig. 1). As the hysterectomy proceeded, an unexpected firm, round, smooth-surfaced mass was visualized in the left lower quadrant. This mass measured roughly 5–6 cm and was found intimately adherent to the serosal surface of the large intestine. Careful inspection demonstrated no remaining anatomic or vascular continuity with the uterus, consistent with a parasitic leiomyoma. The mass received its blood supply from mesenteric vessels, and the surrounding tissue showed evidence of chronic inflammatory adhesion. Given the proximity to the bowel, the surgical team performed meticulous adhesiolysis, employing sharp and blunt dissection techniques to prevent injury to the serosal layer and underlying mesentery (Fig. 2a–d). After full mobilization, the parasitic leiomyoma was separated from the bowel without complication. No bowel perforation, thermal injury, or bleeding was observed. The hysterectomy then proceeded with transection of the uterine vessels, development of the bladder flap, completion of colpotomy, and vaginal delivery of the uterus. The parasitic leiomyoma was delivered through the vaginal cuff immediately afterward. Hemostasis was confirmed, and a cystoscopy was performed, demonstrating bilateral ureteral jets.

### Pathologic evaluation

Gross examination of the uterus revealed a multi-fibroid specimen with several well-circumscribed intramural and subserosal nodules. The parasitic leiomyoma was a separate, smooth, whorled, tan-white mass consistent with benign leiomyoma morphology. Microscopic evaluation showed intersecting bundles of smooth muscle cells without nuclear atypia, coagulative necrosis, or increased mitotic activity. Both fallopian tubes were benign. The pathology was diagnostic of intramural leiomyomas, with a separate parasitic leiomyoma, all of which were without malignant features.

### Histopathology

Benign leiomyomas characteristically show whorled bundles of smooth muscle cells with minimal atypia and low mitotic figures. Distinguishing these from leiomyosarcomas is critical; malignant features include tumor cell necrosis, severe atypia, and brisk mitotic activity [13]. The absence of these features confirms benign pathology.

### Postoperative course

Postoperative recovery was uncomplicated. The patient was discharged home on postoperative day 1 with routine pain control. At the 1-week postoperative visit, she reported improved mobility, resolution of pelvic pressure, and complete cessation of abnormal bleeding.

## Discussion

Parasitic leiomyomas are rare but clinically significant entities that may complicate the surgical management of uterine fibroids. Their extrauterine location and autonomous blood supply contribute to diagnostic difficulty and increase the risk of intraoperative complications [5–7]. Although hysterectomy is typically definitive for symptomatic uterine leiomyomas, parasitic lesions may persist independently and require separate excision.

Complete surgical excision is the treatment of choice and is generally curative [5, 6]. Because parasitic leiomyomas often derive blood supply from nongynecologic structures such as the bowel mesentery or omentum, meticulous identification and control of feeding vessels are essential to prevent hemorrhage or visceral injury [6, 7]. Minimally invasive and robotic-assisted approaches may facilitate safe excision by providing enhanced visualization and precision during adhesiolysis [10]. Parasitic leiomyomas involving the bowel are uncommon but clinically important. Extrauterine leiomyomas adherent to intestinal serosa may exert mass effect, cause chronic inflammatory adhesions, or alter bowel motility, and in rare cases may result in bowel obstruction or ischemia [5–7]. Dense adherence to the bowel increases the risk of inadvertent enterotomy or bleeding during surgical manipulation. Failure to identify parasitic lesions intraoperatively may result in persistent symptoms or delayed complications requiring reoperation [6, 7]. Although bowel resection is rarely necessary, it may be required when lesions compromise bowel integrity or vascular supply [6]. From a molecular perspective, parasitic leiomyomas share biologic features with uterine fibroids. These tumors express estrogen and progesterone receptors and remain hormonally responsive despite detachment from the uterus [14]. Their survival is supported by angiogenic signaling pathways, including upregulation of vascular endothelial growth factor, which promotes neovascularization from surrounding tissues [14, 15]. Alterations in the extracellular matrix and mechanotransduction pathways further contribute to tumor persistence and growth after reimplantation [15]. These molecular mechanisms explain why parasitic leiomyomas may continue to enlarge over time and why medical therapy alone is insufficient once an autonomous vascular supply has been established. Because parasitic leiomyomas are capable of continued growth through hormonal responsiveness and angiogenesis, definitive surgical excision is recommended once identified [5, 6]. When lesions involve bowel or mesentery, robotic-assisted surgery may reduce morbidity by allowing controlled vascular dissection and precise adhesiolysis, potentially avoiding laparotomy or bowel resection in appropriately selected patients [10]. Following complete excision, recurrence appears uncommon, particularly in spontaneous cases not associated with morcellation [6–8]. Symptom-based postoperative surveillance is appropriate, with additional imaging reserved for patients who develop recurrent abdominal or pelvic symptoms.

### Conclusions

Parasitic leiomyomas are rare but clinically important entities that may be encountered unexpectedly during hysterectomy for large fibroid uteri. Their extrauterine location and independent vascular supply can significantly increase surgical complexity and require meticulous dissection to avoid bowel or vascular injury. Awareness of parasitic leiomyomas, along with an understanding of their pathogenesis, molecular biology, and treatment considerations, allows gynecologic surgeons to optimize intraoperative decision-making and improve patient outcomes. Early identification and complete surgical excision are particularly critical when lesions involve bowel structures, as delayed recognition may increase the risk of complications and the need for more extensive surgical intervention.

### Learning points

Parasitic leiomyomas represent a rare manifestation of uterine fibroid disease in which a detached myoma establishes an independent blood supply from adjacent structures, such as the bowel or omentum, resulting in an extra-uterine mass.

Preoperative diagnosis is frequently challenging, as imaging studies may attribute pelvic abnormalities to primary uterine pathology, obscuring identification of an independently vascularized parasitic leiomyoma.

This case highlights a rare parasitic leiomyoma densely adherent to the large intestine that was discovered unexpectedly during a robotic-assisted hysterectomy performed for complex abnormal uterine bleeding.

The coexistence of severe abnormal uterine bleeding and a parasitic leiomyoma poses unique diagnostic and surgical challenges, emphasizing the importance of intraoperative vigilance in patients with long-standing or extensive fibroid disease.

Robotic-assisted minimally invasive surgery allowed safe identification, precise adhesiolysis, and controlled excision of the parasitic lesion while minimizing the risk of bowel or vascular injury.

Recognition of both spontaneous and iatrogenic parasitic leiomyomas is essential, as these lesions represent a rare but clinically significant cause of unexplained pelvic masses in patients with current or prior fibroid disease.

## Data Availability

The authors declare that data supporting the findings of this study are available within the article.

## References

[1] Kho KA, Nezhat C (2009). Parasitic myomas. Obstet Gynecol.

[2] Munro MG, Critchley HO, Fraser IS (2011). The FIGO classification of causes of abnormal uterine bleeding: Malcolm G. Munro, Hilary O.D. Crithcley, Ian S. Fraser, for the FIGO Working Group on Menstrual Disorders. Int J Gynaecol Obstet.

[3] Baird DD, Dunson DB, Hill MC, Cousins D, Schectman JM (2003). High cumulative incidence of uterine leiomyoma in black and white women: ultrasound evidence. Am J Obstet Gynecol.

[4] Fasih N, Prasad Shanbhogue AK, Macdonald DB, Fraser-Hill MA, Papadatos D, Kielar AZ, Doherty GP (2008). Leiomyomas beyond the uterus: unusual locations, rare manifestations. Radiographics.

[5] Laughlin-Tommaso SK, Stewart EA (2018). Moving toward individualized medicine for uterine leiomyomas. Obstet Gynecol.

[6] Kelly HA, Cullen TS (1909). Myomata of the Uterus.

[7] Lete I, Gonzalez J, Ugarte L, Barbadillo N, Lapuente O, Alvarez-Sala J (2016). Parasitic leiomyomas: a systematic review. Eur J Obstet Gynecol Reprod Biol.

[8] Sharma P, Chaturvedi KU, Gupta R (2018). Spontaneous parasitic leiomyoma. J Obstet Gynaecol India.

[9] Kho RM, Abrao MS (2017). Parasitic leiomyomas following laparoscopic surgery. Best Pract Res Clin Obstet Gynaecol.

[10] Uccella S, Cromi A, Bogani G (2016). Parasitic leiomyomas after laparoscopic surgery. J Minim Invasive Gynecol.

[11] Mehsud I, Awan AS, Sabir M (2020). Parasitic leiomyoma mimicking a pelvic mass. Cureus.

[12] Ghezzi F, Raio L, Mueller MD (2006). Radiologic presentation of extrauterine leiomyomas. Obstet Gynecol Clin North Am.

[13] Bell SW, Kempson RL, Hendrickson MR (1994). Problematic uterine smooth muscle neoplasms. A clinicopathologic study of 213 cases. Am J Surg Pathol.

[14] Laughlin-Tommaso SK, Stewart EA (2018). Uterine fibroids and leiomyoma biology. Fertil Steril.

[15] Leppert PC, Jayes FL, Segars JH (2014). The extracellular matrix contributes to mechanotransduction in uterine fibroids. Obstet Gynecol Int.

